# Genetic colocalization atlas points to common regulatory sites and genes for hematopoietic traits and hematopoietic contributions to disease phenotypes

**DOI:** 10.1186/s12920-020-00742-9

**Published:** 2020-06-29

**Authors:** Christopher S. Thom, Benjamin F. Voight

**Affiliations:** 1 Division of Neonatology, Children’s Hospital of Philadelphia, Philadelphia, PA USA; 2 Department of Systems Pharmacology and Translational Therapeutics, University of Pennsylvania - Perelman School of Medicine, Philadelphia, PA USA; 3 Department of Genetics, University of Pennsylvania - Perelman School of Medicine, Philadelphia, PA USA; 4 Institute of Translational Medicine and Therapeutics, University of Pennsylvania - Perelman School of Medicine, Philadelphia, PA USA

**Keywords:** Hematopoiesis, Genetics, Colocalization

## Abstract

**Background:**

Genetic associations link hematopoietic traits and disease end-points, but most causal variants and genes underlying these relationships are unknown. Here, we used genetic colocalization to nominate loci and genes related to shared genetic signal for hematopoietic, cardiovascular, autoimmune, neuropsychiatric, and cancer phenotypes.

**Methods:**

Our aim was to identify colocalization sites for human traits among established genome-wide significant loci. Using genome-wide association study (GWAS) summary statistics, we determined loci where multiple traits colocalized at a false discovery rate < 5%. We then identified quantitative trait loci among colocalization sites to highlight related genes. In addition, we used Mendelian randomization analysis to further investigate certain trait relationships genome-wide.

**Results:**

Our findings recapitulated developmental hematopoietic lineage relationships, identified loci that linked traits with causal genetic relationships, and revealed novel trait associations. Out of 2706 loci with genome-wide significant signal for at least 1 blood trait, we identified 1779 unique sites (66%) with shared genetic signal for 2+ hematologic traits. We could assign some sites to specific developmental cell types during hematopoiesis based on affected traits, including those likely to impact hematopoietic progenitor cells and/or megakaryocyte-erythroid progenitor cells. Through an expanded analysis of 70 human traits, we defined 2+ colocalizing traits at 2123 loci from an analysis of 9852 sites (22%) containing genome-wide significant signal for at least 1 GWAS trait. In addition to variants and genes underlying shared genetic signal between blood traits and disease phenotypes that had been previously related through Mendelian randomization studies, we defined loci and related genes underlying shared signal between eosinophil percentage and eczema. We also identified colocalizing signals in a number of clinically relevant coding mutations, including sites linking *PTPN22* with Crohn’s disease, *NIPA* with coronary artery disease and platelet trait variation, and the hemochromatosis gene *HFE* with altered lipid levels. Finally, we anticipate potential off-target effects on blood traits related novel therapeutic targets, including *TRAIL*.

**Conclusions:**

Our findings provide a road map for gene validation experiments and novel therapeutics related to hematopoietic development, and offer a rationale for pleiotropic interactions between hematopoietic loci and disease end-points.

## Background

Identifying causal loci and genes from human genetic data is integral to elucidating novel disease insights and therapeutic approaches. Quantitative hematopoietic traits are well studied, although relatively few causal variants and genes have been elucidated [1, 2]. Mendelian randomization studies have established causal relationships between hematopoietic traits and cardiovascular, autoimmune and neuropsychiatric disease [2], but causal genes and loci remain elusive.

Genetic colocalization analysis permits identification of shared regulatory loci, with advances extending the scope of potential studies from two to over 10 traits undergoing simultaneous analysis [3–5]. Recently, a colocalization algorithm was used to identify known and novel loci related to cardiovascular traits [5]. Key assumptions of this algorithm include i) consistent linkage disequilibrium patterns across studies (i.e., that studies were conducted on the same population), ii) there being at most one causal variant per genomic region per trait, and iii) that causal variants are directly identified or imputed in all datasets [5]. We reasoned that a similar analytical pipeline could help explain variants and genes underlying hematopoietic and other disease phenotypes. In this way, aggregated summary statistics might be used to specifically target loci with pleiotropic effects on multiple traits, enacted through one or a handful of genes.

Developmental cell types during hematopoiesis, the process that gives rise to all blood lineages, are relatively well mapped. We hypothesized that shared genetic signal impacting traits from multiple blood lineages might nominate genomic loci related to the stem and progenitor cells that spawned those types of blood cells. This approach is orthogonal to prior data that analyzed patterns in accessible chromatin to define genomic locations affecting multiple blood lineages [1]. For example, a shared single nucleotide polymorphism (SNP) related to quantitative variation in platelet, red blood cell (RBC), and white blood cell (WBC) counts might indicate a site or mechanism that is active in hematopoietic stem and progenitor cells (HSCs). SNPs related to platelet and RBC counts, but not WBC count, might reveal loci and related genes for megakaryocyte-erythroid progenitor (MEP) cells. We hypothesized that the directionality of such relationships might help elucidate lineage decisions during hematopoiesis, and help target loci and genes related to developmental hematopoiesis.

Blood traits are related to a number of human disease phenotypes [2]. Blood cells can cause disease (e.g., autoimmune traits) or be affected by therapies (e.g., anemia secondary to chemotherapy). For this reason, understanding pleiotropic associations between blood and other traits could reveal translationally relevant trait relationships or help predict off-target effects of gene-modifying therapies.

Here, we used genetic colocalization to define sites wherein two or more human traits shared genetic signal at genome-wide significant loci. We initially examined blood traits, and later expanded our analysis to include a total of 70 blood, autoimmune, cardiovascular, cancer, and neuropsychiatric traits. We then looked for quantitative trait loci impacting gene expression (eQTL) or exon splicing variation (sQTL) at or near sites of genetic colocalization. Our results identify sites that affect specific cell types during hematopoietic development, and reveal genetic variants underlying trait relationships between blood parameters and disease end-points.

## Methods

### SNP and study selection

GWAS summary statistics were obtained from publicly available repositories (Additional file 1: Table S1 [2, 6–21]). We narrowed analysis to just those GWAS summary statistics for European populations with > 1 × 10^6^ sites (i.e., those that were genome-wide). Analyzed SNPs were identified as genome-wide significant in the largest hematopoietic trait GWAS to date [2] or from a repository of genome-wide significant SNPs from a compilation of GWAS from the NHGRI-EBI Catalog (downloaded January 2019) [22]. In addition, we analyzed quantitative trait locus data from GTEx V7 [23]. Human genome version hg19 was used for all analyses.

### Colocalization analyses

We used the HyPrColoc software to conduct colocalization experiments [5]. This software requires effect (e.g., beta or odds ratio) and standard error values for each analyzed SNP. We chose to analyze based on chromosome and position, given that multiple rsIDs might overlap at a given locus and be inconsistent between different GWAS. Although this removed duplicate rsIDs and may have caused some bias, we reasoned that this would be a minority of sites. This strategy optimized the number of individual positions that we were able to incorporate into our input dataset for colocalization analysis. We specifically looked at 500 kb regions (250 kb on either side of each site), in line with prior colocalization literature [5].

**Fig. 1:**
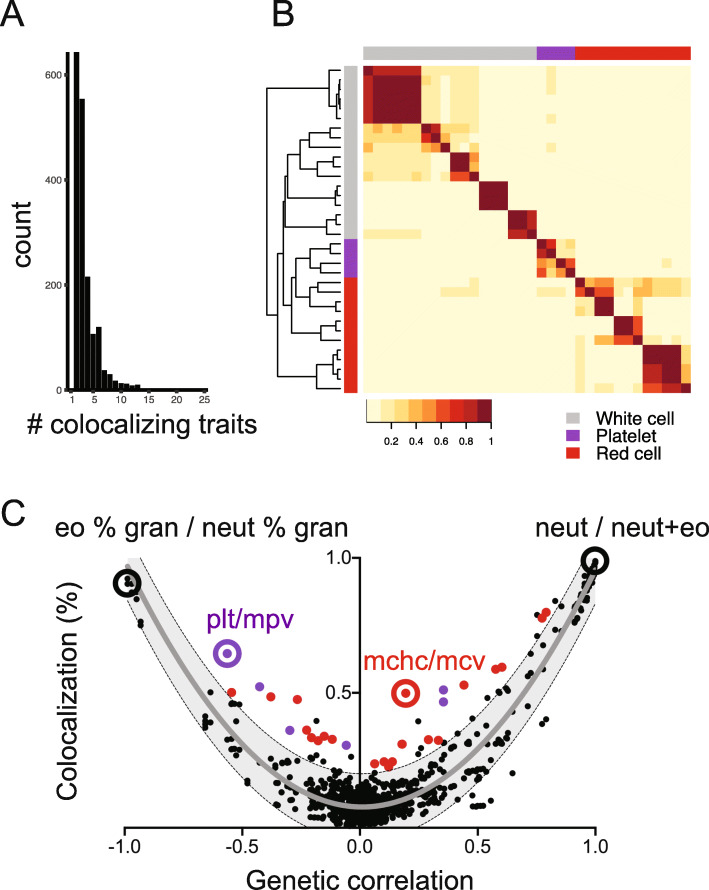
Genetic colocalization between blood traits reflects hematopoietic lineage relationships. **a** Number of traits identified at each colocalization site (max = 25). **b** Heat map depicting percent overlap at colocalization sites between each hematopoietic trait pair. In each box, the number of sites where the row-specified trait and column-specified trait colocalized was normalized to the total number of colocalization sites for the ‘row trait’. For this reason, the heat map is asymmetric. Color scale represents the proportion of loci where each pair of traits colocalized. To the left of the heat map, hierarchical clustering accurately segregated red cell, platelet, and white cell traits in general agreement with blood lineage relationships. **c** Degree of colocalization (% overlap) generally reflects genetic correlation between trait pairs. Shaded area depicts the 95% prediction interval, with gray line at mean. Colored spots highlight trait pairs outside the 95% prediction interval that included two platelet traits (purple) or two red blood cell traits (red). Exemplary trait pairs are circled. Eo % gran, percentage of granulocytes that are eosinophils. Neut % gran, percentage of granulocytes that are neutrophils. Plt, platelet count. Mpv, mean platelet volume. Mchc, mean corpuscular hemoglobin content. Mcv, mean red cell corpuscular volume. Neut, neutrophil count. Neut+eo, total neutrophil plus eosinophil count

**Fig. 2:**
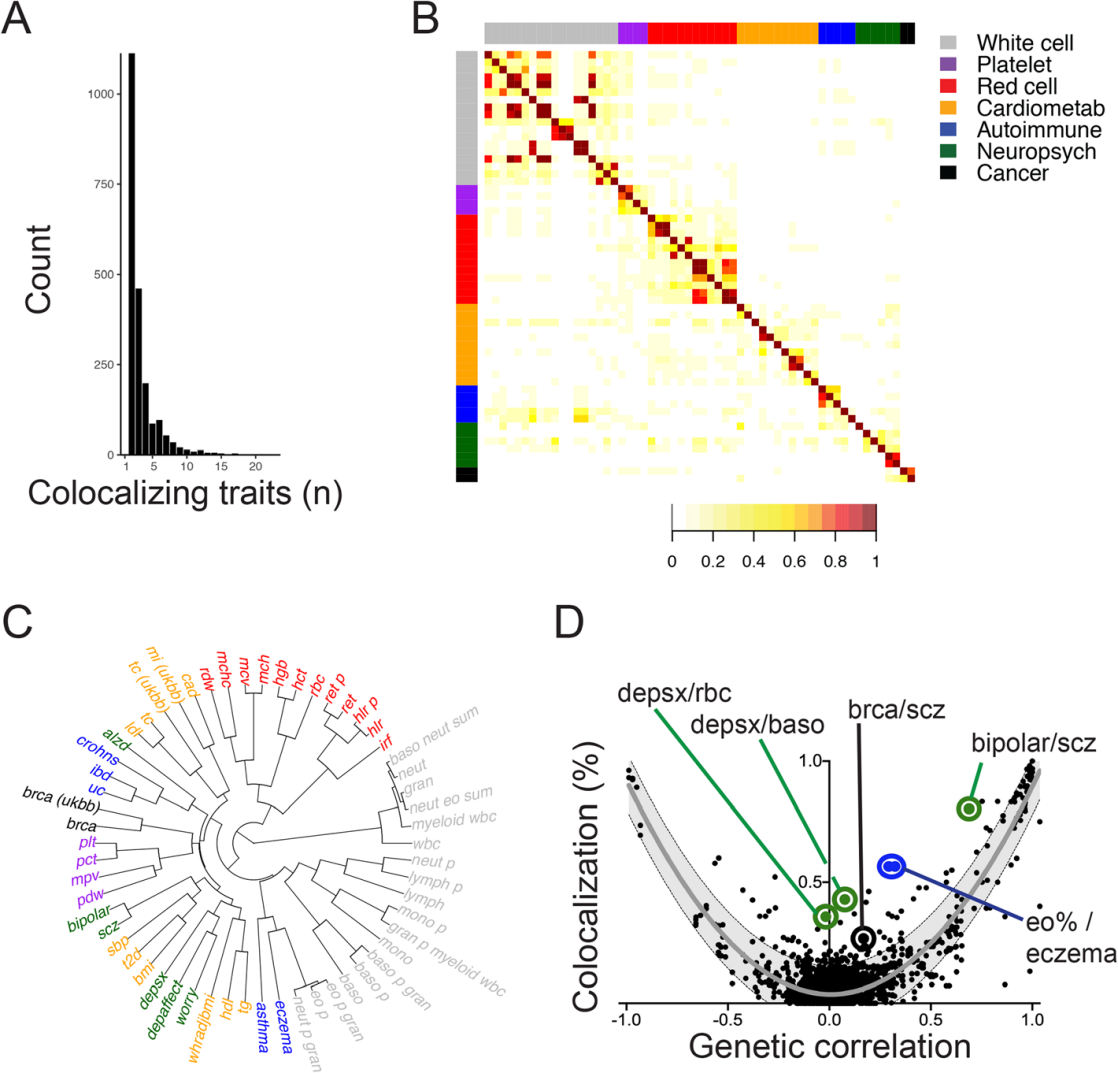
Genetic colocalization reveals shared regulatory loci and implicates causal genes underlying genetic associations between hematopoietic traits and disease end-points. **a** Number of traits identified at each colocalization site (max = 24). **b** Heat map depicting percent overlap at colocalization sites between each trait pair. In each box, the number of sites where the row-specified trait and column-specified trait colocalized was normalized to the total number of colocalization sites for the ‘row trait’. For this reason, the heat map is asymmetric. **c** Hierarchical clustering based on colocalization results associates related traits, which are color coded according to the key in part **b**. **d** Degree of colocalization (% overlap) reflects genetic correlation between trait pairs. Shaded area depicts the 95% prediction interval, with gray line at mean. Exemplary trait pairs are circled. Depsx, depressive symptoms. Rbc, red blood cell count. Baso, basophil cell count. Brca, breast cancer. Scz, schizophrenia. Eo%, eosinophil percentage of white blood cells (‘eo_p’) or granulocytes (‘eo_p_gran’)

As the SNPs considered as input data varied between analyses, we presented separate results from analysis of 34 hematologic traits, and a composite of 70 traits. GWAS summary statistics were harmonized prior to analyses (https://github.com/hakyimlab/summary-gwas-imputation/wiki). There were 29,239,447 genomic sites analyzed for colocalization among the hematologic traits. A total of 1,667,428 harmonized sites were analyzed from GWAS summary statistics for the 70 traits. The decreased number of sites included in this latter analysis resulted in decreased power to detect associations. This was reflected in the maximum number of traits colocalized, which in Fig. 1a was 25 (out of 34 traits) versus 24 traits in Fig. 2a (out of 70 traits). The number of sites used for ‘restricted’ analysis of traits with limited genetic correlation (r_g_ < 0.8) were similar to ‘full’ analyses, including 29,261,510 genomic sites for 17 blood traits (Additional file 2: Figure S1), and 1,667,741 genomic sites for 45 traits (Additional file 2: Figure S2).

After colocalization analysis, we narrowed our focus on only those loci with posterior probability for colocalization (PPFC) > 0.7, based on empiric simulations results from the creators of this algorithm showing that this conservatively gave a false discovery rate < 5% [5]. We noted that a more relaxed PPFC (e.g., > 0.5) yielded substantially more loci. A less conservative threshold could in this way be used as a hypothesis-generating experiment for cellular follow up studies.

### Coding variant identification

We used the Ensembl Variant Effect Predictor (http://grch37.ensembl.org/Homo_sapiens/Tools/VEP) to identify coding variants and related gene consequences.

### Linkage disequilibrium and quantitative trait locus (QTL) analyses

We wanted to assess comprehensively the potential gene expression or splicing changes related to colocalization sites. Thus, we analyzed each colocalization site together with all sites in high linkage disequilibrium (EUR r^2^ > 0.90, PLINK version 1.9).

We used closestBed (https://bedtools.readthedocs.io) to identify the nearest gene to each SNP. Genes and positions were defined by BioMart (https://grch37.ensembl.org/biomart/martview).

For each group of linked SNPs around a colocalization locus, we identified all eQTLs (GTEx V7 [23]), as well as all sQTLs as defined by two different algorithms (GTEx V3 sQTLseekeR [24], Altrans [25]). In the manuscript and Additional file 1, the quantity of QTL SNPs and pathway analyses reflect a composite of all genes impacted by a given locus, or by highly linked SNPs. Note that a given colocalization site might be linked with several SNPs, and that these SNPs might be proximal to and/or impact different genes. Affected genes shown are those with a unique Ensembl gene identifier (ENSG). In some cases, gene names may differ between Nearest Gene, eQTL and sQTL columns given that the underlying analyses were derived from different catalogues.

### Gene ontology analysis

We submitted QTLs associated with specific traits for biological pathway assessment using the Gene Ontology (GO) resource (http://geneontology.org). Statistical significance of GO Biological Process enrichments were assessed using binomial tests and Bonferroni correction for multiple testing. Presented data were those pathways with *p* < 0.05.

### Empirical distribution for expected colocalization counts

We used LDSC to estimate genetic correlation between traits (v1.0.1) [26]. Presented genetic correlation data reflect r_g_ values obtained from LDSC analysis.

### Mendelian randomization

We created genetic instrumental variables from GWAS summary statistics for blood traits [2], eczema [15], and depressive symptoms [20]. To generate instrumental variables, we first identified SNPs common to both exposure and outcome data sets. Using Two-sample MR (v0.5.4 [27]) and R (v3.6.3), we then clumped all genome-wide significant SNPs to identify single nucleotide polymorphisms within independent linkage disequilibrium blocks (EUR r^2^ < 0.01) in 10,000 kb regions.

We used mRnd (http://cnsgenomics.com/shiny/mRnd, [28]) to estimate the F-statistics of our instrumental variables. We calculated the proportion of genetic inheritance explained per Shim *et. al.* [29]. None of our instrumental variables was subject to weak instrument bias, as each had an F-statistic > 10 [28].

#### Data presentation

Data were created and presented using R, Adobe Illustrator CS6 and GraphPad Prism 8.

#### Statistics

Statistical analyses were conducted using R and GraphPad Prism 8.

## Results

### Genetic colocalization recapitulates hematopoietic lineage relationships

Our first aim was to validate whether colocalization could effectively capture known trait relationships and genetic correlations between hematopoietic lineages [1]. We performed colocalization analysis [5] using genome-wide association study (GWAS) summary statistics related to 34 quantitative hematopoietic traits for 2706 genome-wide significant loci [2], revealing a total of 1779 sites wherein 2 or more traits colocalized with a PPFC > 0.7 (Additional file 1: Table S2). In simulations, these criteria identified the causal variant, or a variant in high LD with the causal variant, with a false discovery rate < 5% [5]. Colocalization sites specified 3.6 ± 2.3 traits (mean ± SD), with 22% of the loci (259 in total) representing highly pleiotropic sites where 6 or more traits colocalized (Fig. 1a). Hence, a substantial proportion of interrogated loci (66%) impacted multiple hematopoietic traits.

To investigate trait relationships, we constructed a heat map to depict the percentage colocalization between trait pairs (Fig. 1b). Hierarchical clustering of colocalization results reflected blood lineage relationships, with platelet, erythroid, and white blood cell traits generally clustering as expected.

We then asked whether our colocalization findings mirrored genetic correlation between hematopoietic traits [26]. Indeed, more closely related traits colocalized more often (Fig. 1c, r^2^ = 0.91 by quadratic regression with least squares fit). Directly correlated (e.g., ‘neutrophil count’ and ‘neutrophil + eosinophil count’; ‘granulocyte count’ and ‘myeloid white blood cell count’), and inversely correlated trait pairs (e.g., ‘eosinophil percent of granulocytes’ and ‘neutrophil percent of granulocytes’; ‘lymphocyte percent’ and ‘neutrophil percent’), essentially always colocalized.

Several trait pairs fell outside the 95% prediction interval. The majority of these trait pairs included two traits from the same hematopoietic lineage (e.g., ‘mean platelet volume’ and ‘platelet count’; ‘mean corpuscular hemoglobin concentration’ and ‘mean red cell volume’) (Fig. 1c). Lineage-critical loci or genes might be expected to have more significant influence on these trait pairs than would be captured by genetic correlation measurement.

In sum, these results validated the notion that colocalization analysis results would mirror genetic correlation, and reflect known relationships among hematopoietic lineages and traits. Interestingly, trait pairs without genetic correlation frequently had some degree of colocalization (Fig. 1c, y-intercept = 0.077 ± 0.123). This likely reflects horizontal pleiotropy, in which a given locus and related gene(s) impact traits that are not biologically related. In the context of hematopoietic development, our derived estimate of chance colocalization between unrelated traits is therefore ~ 8%.

Given high genetic concordance between some blood traits, we also performed colocalization analysis after removing traits with genetic correlation (r_g_) > 0.8. This experiment, using 17 quantitative hematopoietic traits, identified 946 colocalization sites for 2 or more traits with a PPFC > 0.7, representing 35% of interrogated loci (Additional file 1: Table S3 and Additional file 2: Figure S1). Compared with our analysis of 34 blood traits, the number of traits that colocalized at each locus was reduced as expected (2.6 ± 1.3, mean ± SD). Importantly, both analyses identified similar sites and trait relationships. Below, we focus on findings from more comprehensive colocalization experiments using 34 traits.

### A genetic colocalization strategy to identify loci related to hematopoietic development

We leveraged our colocalization results to identify quantitative trait loci (QTLs) related to specific hematopoietic lineages and cell types. For example, loci where white blood cell (WBC), red blood cell (RBC), and platelet counts colocalize might indicate developmental perturbation in hematopoietic stem and progenitor cells (HSCs). We therefore looked for sites of colocalization between these quantitative blood traits, and identified overlapping genome-wide significant QTLs. Indeed, QTLs related to these loci pointed to known HSC regulatory genes *SH2B3* [30, 31], *ATM* [32], and *HBS1L-MYB* [33] (Additional file 1: Table S4).

We also parsed loci identified by colocalization to specifically affect platelet or red cell traits, with the hypothesis that these loci would relate to terminally differentiated blood cell biology. There were 439 sites nominated by colocalization analysis specifically for red cell traits (RBC, HCT, MCV, MCH, MCHC, RDW) but *not* platelet traits or WBC count. These sites, or highly linked loci, influenced expression of 614 genes (123 genes in whole blood, Additional file 1: Table S5). Among genes regulated in whole blood were *RHD* [34]*, HBZ* [35], and *LPL* [36], which can influence erythroid stability and/or lifespan, as well as *SP1* [37]*, ESR2* [38], and *FANCA* [39], which impact erythropoiesis. Gene ontology (GO) analysis [40] of these gene sets revealed significant enrichment of genes related to cellular metabolic processes (Additional file 1: Table S6). A similar analysis of platelet trait-restricted sites (PLT, PCT, MPV, PDW), including highly linked loci, identified 270 sites impacting expression of 399 genes (77 genes in whole blood, Additional file 1: Table S7). These genes included *STIM1* [41] and *C4BPA* [42], which impact platelet reactivity and/or thrombosis risk, as well as *MASTL* [43] and *TPM4* [44], which influence megakaryo-thrombopoiesis. Pathway analysis of these genes revealed enrichment of apoptotic cell clearance and metabolic processes (Additional file 1: Table S8). Complement-mediated apoptotic cell clearance mechanisms are indeed important for regulating platelet count [45].

To our surprise, pathways analyses of red cell and platelet lineage-restricted colocalization QTLs were not enriched for processes ascribed to hematopoiesis, erythropoiesis, or megakaryopoiesis. This suggests that genes and processes linked to terminal red cell and platelet traits are largely impacted by cellular function and reactivity, rather than developmental perturbations. With notable exceptions whereby causal loci do impact hematopoietic development (e.g., [30, 46–48]), our findings suggest the many of the identified genes and factors may not impact hematopoiesis per se. In fact, our results indicate that blood cell-extrinsic properties (e.g., apoptotic cell clearance mechanisms) frequently impact quantitative hematopoietic traits. In sum, our findings reveal a multitude of known variants and genes, as well as novel QTLs and related genes that warrant further study.

### Illuminating hematopoietic contributions and associations with disease phenotypes

We then applied an extended colocalization analysis to summary statistics for 70 total hematopoietic, cardiovascular, autoimmune, cancer, and neuropsychiatric traits (Additional file 1: Table S1 [2, 6–21]). Variations in size and power across these studies would be expected to influence detection of trait associations and/or colocalizations. Following allele harmonization, colocalization analysis using 9852 genome-wide significant loci from the NHGRI-EBI database [22] and blood traits [2] revealed a total of 2123 sites (22%) wherein two or more traits colocalized with a PPFC > 0.7 (Additional file 1: Table S9). The average number of traits that colocalized at a given site was 3.3 ± 2.4 (mean ± SD), with 83 loci identified as a ‘very pleiotropic’ colocalization site for ≥9 traits (Fig. 2a). Known trait relationships were recapitulated among these colocalization sites (e.g. bipolar disorder and schizophrenia; Fig. 2b-d). These results again reflected genetic correlation between traits, estimating a small degree of pleiotropy (~ 4%) absent genetic correlation (Fig. 2d, r^2^ = 0.83, y-intercept = 0.037 ± 0.117).

Restricted analysis of 45 traits with genetic correlation (r_g_) < 0.8 identified 1670 colocalization sites, with 2.6 ± 1.3 (mean ± SD) colocalizing traits per locus (Additional file 1: Supplemental Table S10 and Additional file 2: Figure S2). This latter experiment identified similar loci and trait relationships as our 70-trait analysis. In the interest of providing as comprehensive a list of results as possible, the findings discussed below are derived from joint analysis of 70 traits.

Mendelian randomization analyses, which use genetic variants to estimate causal effects of a genetically-determined exposure of interest on an outcome, have established relationships between blood traits and some disease phenotypes [2]. Despite holding significant therapeutic potential, most causal loci and genes underlying these associations are unknown. Our results reveal putatively causal loci, related genes, and molecular pathways related to these trait pairs (Additional file 1: Tables S11-S17). For example, whole blood QTLs related to genes known to affect asthma pathogenesis or severity (e.g. *IL18R* [49–51]*, ZFP57* [52]*, BTN3A2* [53]*, NDFIP1* [54]*, SMAD3* [55]*, CLEC16A* [56]*,* and *TSLP* [57]) were associated with colocalization sites for asthma and neutrophil, eosinophil, monocyte and/or lymphocyte traits (Additional file 1: Tables S11-S14). Similarly, QTLs for genes linked to coronary artery disease risk (e.g. *LPL* [58]*, SREBF1* [59]*, GIT1* [60, 61]*, SKIV2L* [61]*, MAP3K11/MLK3* [62]) were associated with colocalization sites linking coronary artery disease with mean platelet volume, lymphocyte, and/or reticulocyte counts (Additional file 1: Tables S15-S17). Other identified genes associated with colocalization loci represent novel findings that could enhance understanding of the pathophysiology and/or treatment of these diseases, although functional validation remains necessary.

Our findings also revealed novel trait associations. For example, eosinophil percentage and eczema colocalized more often than predicted based on their genetic correlation (Fig. 2d). These traits are clinically related [63] and colocalized at 13 loci (Additional file 1: Table S18), including sites near genes that regulate eosinophil biology (*ETS1* [64, 65] and *ID2* [65]) and autoimmune disease (*KIAA1109* [66–68] and *TAGAP* [69]). These colocalization sites also indicated potential regulation of unexpected genes that warrant validation (*SNX32, ZNF652, KLC2*).

We reasoned that Mendelian randomization analysis might provide additional support for this trait relationship. However, we did not necessarily expect significant genome-wide association, given that our colocalization analysis highlighted a fairly restricted subset of loci. By Mendelian randomization, we identified a 27% increased risk of eczema for each 1 standard deviation increase in eosinophil percentage by inverse variance weighted method (95% confidence interval = 9–48%, *P* = 0.002), although the association did not reach statistical significance for weighted median or MR-Egger methods (Additional file 1: Tables S19-S20). This analysis did not show evidence of horizontal pleiotropy (MR-Egger intercept *P* = 0.87) and the instrumental variable was not subject to weak instrument bias (F-statistic = 132, Additional file 1: Table S19) [28]. Although these findings would not constitute strong independent evidence of causality alone, they did lend some additional support to the relationship identified through colocalization analysis.

We also identified 5 colocalization sites for red blood cell count, basophil count, and depressive symptoms, which exceeded expectations based on genetic correlation (Fig. 2d and Additional file 1: Table S21). These colocalization sites included eQTLs for *YPEL3*, which is highly expressed in whole blood [23] and affects neural development [70], as well as *PRSS16*, which impacts immunologic development [71] and has been implicated in multiple GWAS for depression phenotypes [72]. While blood phenotypes may impact depressive symptoms, it is also possible that these eQTLs and genes have separate functions in hematopoietic and brain tissues. Mendelian randomization experiments did not identify statistically significant causal relationships for red blood cell count or basophil count on depressive symptoms (Additional file 1: Table S19 and Additional file 1: Tables S22-S23), consistent with low genome-wide correlation (Fig. 2d). Future GWAS for depressive symptoms with increased size and power may better elucidate causal relationships, if such relationships exist.

In addition, we identified trait relationships beyond hematologic parameters, including 4 colocalization sites for breast cancer and schizophrenia (Fig. 2d and Additional file 1: Supplemental Table S24). Recent epidemiologic [73] and genetic [74] studies have linked schizophrenia and breast cancer risks. Our results nominate *TCF7L2* [75, 76]*, BCAR1* [77], and *NEK10* [78, 79] as potential targets to help explain this association.

### Colocalization at coding variation sites identifies clinically relevant trait associations

We reasoned that colocalizing sites could help explain unexpected or pleiotropic effects of gene perturbations. Here, we focused on missense variation in coding regions to establish direct locus-gene relationships. This approach identified clinically relevant cross-trait associations.

Variation in rs2476601 causes a missense mutation in *PTPN22* (Cys1858Thr). This site has been linked to autoimmunity and Crohn’s disease phenotypes, but not ulcerative colitis [80]. Immune response dysregulation, including WBC biology, contributes to the Crohn’s phenotype [80]. We identified shared genetic signal for increased Crohn’s disease risk and decreased WBC count, but not ulcerative colitis, at this location (Additional file 1: Table S25). This finding supports a specific clinical association with Crohn’s disease for the *PTPN22* Cys1858Thr mutation.

Mean platelet volume (MPV) variation has previously been linked to altered risk of coronary artery disease, but understanding of genes underlying this association is lacking [2]. We identified colocalizing signals for increased coronary artery disease risk and increased MPV in a missense coding mutation for *ZC3HC1/NIPA* (Additional file 1: Table S15). This variant causes an Arg > His missense change in several *NIPA* isoforms. *NIPA* impacts heart disease risk and cell cycle regulation [81]. Further studies are needed to understand how this gene might coordinately impact platelet biology and coronary artery disease risk, as well as other traits linked to this locus.

Altered lipid and cholesterol levels have been clinically observed in patients with hereditary hemochromatosis due to mutations in *High FE*^*2+*^ (‘high iron’, *HFE*) [82]. Patients with hemochromatosis have lower cholesterol levels than normal, although an open question is whether this observation is due to manifestations of disease or *HFE* deficiency itself. Our data show that individuals heterozygous for the Cys282Tyr allele have lower reticulocyte count and higher total cholesterol and low density lipoprotein levels (Additional file 1: Table S26). This suggests that *HFE* haploinsufficiency increases cholesterol and lipid levels, and that decreased cholesterol in patients with hemochromatosis occurs secondary to myriad tissue manifestations of clinically significant hemochromatosis or iron overload [83].

Finally, we hypothesized that our analysis might also help predict off-target effects of novel therapeutic agents. For example, tumor necrosis factor (TNF)-related apoptosis inducing ligand (*TRAIL*) is a promising novel chemotherapeutic target [84]. A mutation in the *TRAIL* 3′ UTR was recently associated with decreased triglyceride levels [85]. Targeted analysis of this site identified colocalizing signals for altered myeloid and platelet indices (Additional file 1: Table S27). It will be interesting to see whether these traits are affected in upcoming clinical trials targeting *TRAIL*.

## Discussion

Genetic colocalization approaches have proven a powerful tool in revealing pleiotropic effects of certain loci on multiple traits [3, 4]. Here, we have adapted the colocalization methodology to reveal sites and genes related to specific cell stages in hematopoietic development, and identify relevant trait relationships between blood traits and human disease end-points. We present what we believe to be a minimal estimate of these associations, given the assumption of at most one causal locus per genomic region and our conservative threshold for colocalization (PPFC > 0.7). This threshold revealed high-confidence targets, although future gene discovery studies might instead use a more relaxed threshold (e.g., PPFC > 0.5) to enable a more encompassing set of loci.

GWAS have linked thousands of genomic sites with blood trait variation [2]. The biology related to each site could relate to developmental hematopoiesis, as has been shown for *CCND3* [46]*, CCNA2* [47]*, SH2B3* [30], and *RBM38* [48]. Alternatively, biology related to GWAS sites might impact terminally differentiated cell reactivity or turnover. For example, altered platelet reactivity can affect quantitative platelet traits [86, 87]. Cellular validation experiments might be streamlined if one could better parse relevant sites, genes and developmental stages based on GWAS information. Gene targets presented herein represent one approach to such a computational pipeline, and are orthogonal to previously published findings based on accessible chromatin patterns during hematopoietic development [1]. Future studies combining these computational modalities might be useful for those interested in evaluating specific genes or loci in blood progenitor biology.

Our expanded analysis of 70 human traits recapitulated known trait relationships between blood traits and human disease phenotypes, and identified sites with potential translational relevance. Variations in GWAS size and power may have limited our ability to identify certain trait associations. We anticipate that increasingly well-powered GWAS will likely to expand the catalogue of colocalizations in the future. Larger studies may also reveal new causal genetic associations in Mendelian randomization analyses, although trait relationships need not meet genome-wide significance to be biologically important. In fact, each colocalization site identified in our analysis could be viewed as a hypothesis-generating site for future cellular validation. Understanding trait relationships through colocalization analysis may also be useful for multivariable Mendelian randomization and/or mediation analyses designed to reveal causal biological mechanisms.

Understanding how missense coding mutations impact phenotypes offers the most direct relationship between genes and traits. An adaptation of our colocalization strategy might be employed to predict off-target effects of gene modulation, help understand the cellular basis of disease, or investigate unexpected cellular developmental relationships (e.g., sites related to multiple mesoderm-derived tissues might triangulate to early mesodermal biology). We anticipate an expanded array of such targets could be revealed with larger, trans-ethnic GWAS.

## Conclusion

In an extensive genetic colocalization analysis, we have identified loci, genes and related pathways related to hematopoietic development. Further, our colocalization results identified loci relating 70 hematopoietic, cardiovascular, autoimmune, neuropsychiatric and cancer phenotypes. This repository of associations will be useful for mechanistic studies aimed at understanding biological links between phenotypes, for developing novel therapeutic strategies, and for predicting off-target effects of small molecule and gene therapies.

## Supplementary information

**Additional file 1: Table S1.** Genome wide association study summary statistics used in our analysis. The trait(s) queried are shown, along with study Pubmed identification number (PMID). UK Biobank studies can be found using the link provided. Total European sample sizes, including cases and controls where appropriate, are shown for each study. PMIDs for data downloaded from the NHGRI-EBI GWAS Catalog can be found at the bottom of this table. **Table S2.** Traits and SNPs identified by colocalization analysis [5] of 34 hematopoietic traits. All identified sites are shown in this table. Candidate SNPs are indicated as chr:pos. The posterior probability of colocalization, regional (genomic) probability of colocalization, and posterior probability explained at each locus are indicated. **Table S3.** Traits and SNPs identified by colocalization analysis [5] of 17 hematopoietic traits with genetic correlation (r_g_) < 0.8. All identified sites are shown in this table. Candidate SNPs are indicated as chr:pos. The posterior probability of colocalization, regional (genomic) probability of colocalization, and posterior probability explained at each locus are indicated. **Table S4.** ‘Hematopoietic stem cell’ sites at which white blood cell (wbc), red blood cell (rbc), and platelet (plt) counts colocalize. Sites were specified by chromosome and position. The rsID(s) associated with each site are shown. Gene symbols for the nearest gene, all eQTLs, all eQTLs in whole blood, and all sQTLs (sQTLseekeR and Altrans methods) are shown in the indicated columns related to the indicated SNP (rsID) and any SNPs in high linkage disequilibrium (r^2^ > 0.9). **Table S5.** ‘RBC trait only’ sites at which only the indicated red blood cell traits colocalized, excluding platelet or white blood cell traits. Gene symbols for the nearest gene, all eQTLs, all eQTLs in whole blood, and all sQTLs (sQTLseekeR and Altrans methods) are shown in the indicated columns related to the indicated SNP (rsID) and any SNPs in high linkage disequilibrium (r^2^ > 0.9). Rbc, red blood cell count. Hct, hematocrit. Mcv, mean red cell corpuscular volume. Rdw, red cell distribution width. **Table S6.** Gene ontology pathway analysis of genes regulated by eQTLs linked to ‘RBC trait only’ sites. Shown are pathways with *p* < 0.05 by Binomial test using Bonferroni correction for multiple testing. **Table S7.** ‘Platelet trait only’ sites at which only the indicated platelet traits colocalized, excluding red blood cell or white blood cell traits. Gene symbols for the nearest gene, all eQTLs, all eQTLs in whole blood, and all sQTLs (sQTLseekeR and Altrans methods) are shown in the indicated columns related to the indicated SNP (rsID) and any SNPs in high linkage disequilibrium (r^2^ > 0.9). Plt, platelet count. Pct, platelet-crit. Mpv, mean platelet volume. Pdw, platelet distribution width. **Table S8.** Gene ontology pathway analysis of genes regulated by eQTLs linked to ‘platelet trait only’ sites. Shown are pathways with p < 0.05 by Binomial test using Bonferroni correction for multiple testing. **Table S9.** Traits and SNPs identified by colocalization analysis [5] of 70 human traits. All identified sites are shown in this table. Candidate SNPs are indicated as chr:pos. The posterior probability of colocalization, regional (genomic) probability of colocalization, and posterior probability explained at each locus are indicated. **Table S10.** Traits and SNPs identified by colocalization analysis [5] of 45 human traits with genetic correlation (r_g_) < 0.8. All identified sites are shown in this table. Candidate SNPs are indicated as chr:pos. The posterior probability of colocalization, regional (genomic) probability of colocalization, and posterior probability explained at each locus are indicated. **Table S11.** Colocalization sites for lymphocyte count (lymph) and Asthma. Gene symbols for the nearest gene, all eQTLs, all eQTLs in whole blood, and all sQTLs (sQTLseekeR and Altrans methods) are shown in the indicated columns related to the indicated SNP (rsID) and any SNPs in high linkage disequilibrium (r^2^ > 0.9). **Table S12.** Colocalization sites for neutrophil count (neut) and Asthma. Gene symbols for the nearest gene, all eQTLs, all eQTLs in whole blood, and all sQTLs (sQTLseekeR and Altrans methods) are shown in the indicated columns related to the indicated SNP (rsID) and any SNPs in high linkage disequilibrium (r^2^ > 0.9). **Table S13.** Colocalization sites for eosinophil percentage of white blood cells (eo%) and Asthma. Gene symbols for the nearest gene, all eQTLs, all eQTLs in whole blood, and all sQTLs (sQTLseekeR and Altrans methods) are shown in the indicated columns related to the indicated SNP (rsID) and any SNPs in high linkage disequilibrium (r^2^ > 0.9). **Table S14.** Colocalization sites for monocyte count (mono) and Asthma. Gene symbols for the nearest gene, all eQTLs, all eQTLs in whole blood, and all sQTLs (sQTLseekeR and Altrans methods) are shown in the indicated columns related to the indicated SNP (rsID) and any SNPs in high linkage disequilibrium (r^2^ > 0.9). **Table S15.** Colocalization sites for mean platelet volume (mpv) and coronary artery disease (cad). Gene symbols for the nearest gene, all eQTLs, all eQTLs in whole blood, and all sQTLs (sQTLseekeR and Altrans methods) are shown in the indicated columns related to the indicated SNP (rsID) and any SNPs in high linkage disequilibrium (r^2^ > 0.9). **Table S16.** Colocalization sites for reticulocyte count (ret) and coronary artery disease (cad). Gene symbols for the nearest gene, all eQTLs, all eQTLs in whole blood, and all sQTLs (sQTLseekeR and Altrans methods) are shown in the indicated columns related to the indicated SNP (rsID) and any SNPs in high linkage disequilibrium (r^2^ > 0.9). **Table S17.** Colocalization sites for lymphocyte count (lymph) and coronary artery disease (cad). Gene symbols for the nearest gene, all eQTLs, all eQTLs in whole blood, and all sQTLs (sQTLseekeR and Altrans methods) are shown in the indicated columns related to the indicated SNP (rsID) and any SNPs in high linkage disequilibrium (r^2^ > 0.9). **Table S18.** Colocalization sites for eosinophil percentage of white blood cells (eo%) and Eczema. Gene symbols for the nearest gene, all eQTLs, all eQTLs in whole blood, and all sQTLs (sQTLseekeR and Altrans methods) are shown in the indicated columns related to the indicated SNP (rsID) and any SNPs in high linkage disequilibrium (r^2^ > 0.9). **Table S19.** Mendelian randomization analysis results for the indicated exposure and outcome traits. Outcomes reflect increased risk of eczema (odds ratio) or depressive symptoms (in standard deviation units) per 1 standard deviation increase in exposure by inverse variance weighted, weighted median, and MR-Egger methods. Factors used to calculate genetic variance explained (R^2^ [29]) and instrument strength (F-statistics [28]) are shown to the right of the primary results. Instruments with F-statistics > 10 were considered devoid of weak instrument bias [28]. **Table S20.** Instrumental variable data for MR experiments estimating effects of eosinophil percentage of white blood cells (eo%) on Eczema. The rsID (hg19), chromosome, position, effect allele, other (non-effect) allele, effect sizes and standard errors are shown for each SNP. **Table S21.** Colocalization sites for red blood cell count (rbc), basophil cell count (baso) and depressive symptoms (DepSx). Gene symbols for the nearest gene, all eQTLs, all eQTLs in whole blood, and all sQTLs (sQTLseekeR and Altrans methods) are shown in the indicated columns related to the indicated SNP (rsID) and any SNPs in high linkage disequilibrium (r^2^ > 0.9). **Table S22.** Instrumental variable data for MR experiments estimating effects of red blood cell count (rbc) on depressive symptoms (DepSx). The rsID (hg19), chromosome, position, effect allele, other (non-effect) allele, effect sizes and standard errors are shown for each SNP. **Table S23.** Instrumental variable data for MR experiments estimating effects of basophil cell count (baso) on depressive symptoms (DepSx). The rsID (hg19), chromosome, position, effect allele, other (non-effect) allele, effect sizes and standard errors are shown for each SNP. **Table S24.** Colocalization sites for breast cancer and schizophrenia. Gene symbols for the nearest gene, all eQTLs, all eQTLs in whole blood, and all sQTLs (sQTLseekeR and Altrans methods) are shown in the indicated columns related to the indicated SNP (rsID) and any SNPs in high linkage disequilibrium (r^2^ > 0.9). **Table S25.** Analysis of a coding variant (rs2476601) that causes a missense mutation in *PTPN22* shows significant colocalization with white blood cell (wbc) count and Crohn’s disease. The effect sizes and direction (+/−) are shown. **Table S26.** Colocalization analysis for a coding variant (rs1800562) in *HFE*, mutations in which cause hereditary hemochromatosis. Effects on total cholesterol (TC), low density lipoprotein (LDL), and red blood cell traits (high light scatter reticulocyte count, hlr; high light scatter reticulocyte percentage, hlr_p; mean corpuscular hemoglobin concentration, mchc; red cell distribution width, rdw; reticulocyte count, ret; reticulocyte percentage, ret_p), with significant colocalization signal at this locus, are shown. **Table S27.** Colocalization analysis for a coding variant (rs17600346) in Tumor necrosis factor (TNF)-related apoptosis inducing ligand (*TRAIL*, also known as *TNF10*), based on targeted analysis of the 50 kb region surrounding this site. Effects on colocalized white blood cell (granulocyte percentage of myeloid white blood cells, gran_p_myeloid_wbc; monocyte percentage, mono_p) and platelet traits (platelet-crit, pct; platelet count, plt) are shown.

**Additional file 2: Figure S1.** Colocalization between blood traits with limited genetic relatedness reflects hematopoietic lineage relationships. The 17 traits analyzed were pruned for genetic correlation (r_g_) < 0.8. **a** Number of traits identified at each colocalization site (max = 12). **b** Heat map depicting percent overlap at colocalization sites between each hematopoietic trait pair. In each box, the number of sites where the row-specified trait and column-specified trait colocalized was normalized to the total number of colocalization sites for the ‘row trait’. For this reason, the heat map is asymmetric. Color scale represents the proportion of loci where each pair of traits colocalized. To the left of the heat map, hierarchical clustering accurately segregated red cell, platelet, and white cell traits in general agreement with blood lineage relationships. **c** Degree of colocalization (% overlap) generally reflects genetic correlation between trait pairs. Shaded area depicts the 95% prediction interval, with gray line at mean. Colored spots highlight trait pairs outside the 95% prediction interval that included 2 platelet traits (purple) or 2 red blood cell traits (red). Exemplary trait pairs are labeled. Plt, platelet count. Mpv, mean platelet volume. Pdw, platelet distribution width. Rdw, red blood cell distribution width. Mchc, mean corpuscular hemoglobin content. Mcv, mean red cell corpuscular volume. **Figure S2.** Genetic colocalization among traits with limited genetic correlation reveals shared regulatory loci and implicates causal genes underlying genetic associations between hematopoietic traits and disease end-points. The 45 traits analyzed were pruned for genetic correlation (r_g_) < 0.8. **a** Number of traits identified at each colocalization site (max = 14). **b** Heat map depicting percent overlap at colocalization sites between each trait pair. In each box, the number of sites where the row-specified trait and column-specified trait colocalized was normalized to the total number of colocalization sites for the ‘row trait’. For this reason, the heat map is asymmetric. **c** Hierarchical clustering based on colocalization results associates related traits, which are color coded according to the key in part **b**. **d** Degree of colocalization (% overlap) reflects genetic correlation between trait pairs. Shaded area depicts the 95% prediction interval, with gray line at mean. Exemplary trait pairs are circled. Depsx, depressive symptoms. Rbc, red blood cell count. Baso, basophil cell count. Brca, breast cancer. Scz, schizophrenia. eo%, eosinophil percentage of white blood cells.

## Data Availability

GWAS summary statistics analyzed in the current study were obtained from the references detailed in Additional file 1: Table S1 [2, 6–21], including data for blood traits (www.bloodcellgenetics.org) [2]; type 2 diabetes (http://diagram-consortium.org/downloads.html) [6]; waist-hip ratio-adjusted body mass index (https://portals.broadinstitute.org/collaboration/giant/index.php/GIANT_consortium_data_files) [7]; inflammatory bowel disease, Crohn’s disease, and ulcerative colitis (ftp://ftp.sanger.ac.uk/pub/project/humgen/summary_statistics/human/2016-11-07) [8]; bipolar disorder (https://www.med.unc.edu/pgc/download-results) [9]; cancer and cardiovascular traits from the UK Biobank (http://www.nealelab.is/uk-biobank) [10]; asthma (https://www.ebi.ac.uk/gwas/publications/29273806) [11]; migraine (http://www.headachegenetics.org/content/datasets-and-cohorts) [12]; lipids (http://csg.sph.umich.edu/willer/public/lipids2013) [13]; body mass index (https://portals.broadinstitute.org/collaboration/giant/index.php/GIANT_consortium_data_files) [14]; eczema (https://data.bris.ac.uk/data/dataset/28uchsdpmub118uex26ylacqm) [15]; breast cancer (http://bcac.ccge.medschl.cam.ac.uk/bcacdata/oncoarray/gwas-icogs-and-oncoarray-summary-results) [16]; Alzheimer’s disease (https://ctg.cncr.nl/software/summary_statistics) [17]; neuroticism, worry, and depressive affect (https://ctg.cncr.nl/software/summary_statistics) [18]; schizophrenia (https://www.med.unc.edu/pgc/download-results) [19]; depressive symptoms (https://www.thessgac.org/data) [20]; and coronary artery disease (ftp://ftp.ebi.ac.uk/pub/databases/gwas/summary_statistics/vanderHarstP_29212778_GCST005194) [21]. Genome-wide significant SNPs were compiled from blood trait GWAS summary statistics [2] and the NHGRI-EBI GWAS Catalog v1.0 (https://www.ebi.ac.uk/gwas/docs/file-downloads, see Additional file 1: Table S1 for related study accession numbers) [22]. Quantitative trait locus data were obtained from GTEx (http://www.gtexportal.org/home/datasets) [23]. Human genome version hg19 (GRCh37) was produced and managed by the Genome Reference Consortium (https://www.ncbi.nlm.nih.gov/grc/human). Gene locations were obtained from BioMart (https://grch37.ensembl.org/biomart/martview). Scripts and data sets that were generated and analyzed during the current study are available on GitHub (https://github.com/thomchr/2019.Coloc) and from the corresponding author upon reasonable request.

## Abbreviations

GWAS: Genome-wide association study
SNP: Single nucleotide polymorphism
RBC: Red blood cell (count)
WBC: White blood cell (count)
HSC: Hematopoietic stem cell
MEP: Megakaryocyte-erythroid progenitor cell
eQTL: Expression quantitative trait locus
sQTL: Splicing quantitative trait locus
NHGRI-EBI: National Human Genome Research Institute – European Bioinformatics Institute
GTEx: Genome-Tissue expression project
HyPrColoc: Hypothesis Prioritisation in multi-trait Colocalization
PPFC: Posterior probability for colocalization
ENSG: Ensembl gene identifier
GO: Gene Ontology database
LDSC: Linkage disequilibrium score regression
SD: Standard deviation
Cys: Cysteine
Thr: Threonine
Arg: Arginine
His: Histidine
Tyr: Tyrosine
TNF: Tumor necrosis factor
UTR: Untranslated region
PMID: Pubmed identification number
baso: basophil count
baso_neut_sum: basophil plus neutrophil count sum
baso_p_gran: basophil percentage of granulocytes
baso_p: basophil percentage of white blood cells
eo_p_gran: eosinophil percentage of granulocytes
eo_p: eosinophil percentage of white blood cells
gran: granulocyte count
gran_p_myeloid_wbc: granulocyte percentage of myeloid white blood cells
hct: hematocrit
hgb: hemoglobin
hlr: high light scatter reticulocytes
hlr_p: high light scatter reticulocyte percentage
irf: immature reticulocyte fraction
lymph: lymphocyte count
lymph_p: lymphocyte percentage
mchc: mean corpuscular hemoglobin concentration
mch: mean corpuscular hemoglobin
mcv: mean red cell volume
mono: monocyte count
mono_p: monocyte percentage of white blood cells
mpv: mean platelet volume
myeloid_wbc: myeloid white blood cell count
neut_eo_sum: neutrophil plus eosinophil count sum
neut: neutrophil count
neut_p_gran: neutrophil percentage of granulocytes
neut_p: neutrophil percentage of white blood cells
pct: platelet-crit
pdw: platelet distribution width
plt: platelet count
rdw: red cell distribution width
ret.: reticulocyte count
ret_p: reticulocyte percentage
t2d: type 2 diabetes
ukbb: United Kingdom Biobank
mi: myocardial infarction
chf: congestive heart failure
sbp: systolic blood pressure
whradjbmi: waist-hip ratio adjusted body mass index
uc: ulcerative colitis
ibd: inflammatory bowel disease
cad: coronary artery disease
hdl: high density lipoprotein
tc: total cholesterol
tg: triglyceride level
ldl: low density lipoprotein
bmi: body mass index
scz: schizophrenia
bipolar: bipolar disorder
brca: breast cancer
lungca: lung cancer
skinca: skin cancer
ovca: ovarian cancer
utendometca: uterine and endometrial cancer
prostateca: prostate cancer
testicca: testicular cancer
cll: chronic lymphocytic leukemia
thyroidca: thyroid cancer
alzd: Alzheimer’s disease
depaffect: depressive affect
depsx: depressive symptoms

## Gene name

*PTPN22*: Protein tyrosine phosphatase, non-receptor type 22
*ZC3HC1/NIPA*: Zinc finger C3HC-type protein 1 / Nuclear-interacting partner of ALK
*HFE*: High FE2+
*TRAIL*: TNF-related apoptosis inducing ligand
*SH2B3*: SH2B adaptor protein 3 (also known as LNK)
*ATM*: Ataxia-telangiectasia mutated
*HBS1L*: HBS1 like translational GTPase
*MYB*: Myeloblastosis (transcription factor)
*RHD*: Rh blood group D antigen
*HBZ*: Hemoglobin subunit zeta
*LPL*: Lipoprotein lipase
*SP1*: Specificity protein 1 (transcription factor)
*ESR2*: Estrogen receptor 2
*FANCA*: Fanconi anemia complementation group A
*STIM1*: Stromal interaction molecule 1
*C4BPA*: Complement component 4 binding protein alpha
*MASTL*: Microtubule associated serine/threonine kinase like
*TPM4*: Tropomyosin 4
*IL18R*: Interleukin-18 receptor
*ZFP57*: Zinc finger protein 57 homolog
*BTN3A2*: Butyrophilin subfamily 3 member A2
*NDFIP*: Nedd4 family interacting protein 1
*SMAD3*: SMAD family member 3
*CLEC16A*: C-Type lectin domain containing 16A
*TSLP*: Thymic stromal lymphopoietin
*SREBF*: Sterol regulatory element binding transcription factor 1
*GIT1*: G protein-coupled receptor kinase-interacting protein 1
*SKIV2L*: Ski2 like RNA helicase
*MAP3K11/MLK3*: Mitogen-activated protein kinase kinase kinase 11 / Mixed-lineage protein kinase 3
*ETS1*: ETS proto-oncogene 1, transcription factor
*ID2*: Inhibitor of DNA binding 2
*KIAA1109*: KIAA1109 gene
*TAGAP*: T cell activation RhoGTPase activating protein
*SNX3*: Sorting nexin-3
*ZNF652*: Zinc finger protein 652
*KLC2*: Kinesin light chain 2
*YPEL3*: Yippee like 3
*PRSS16*: Thymus-specific serine protease (also known as Serine protease 16)
*TCF7L2*: Transcription factor 7-like 2
*BCAR1*: Breast cancer anti-estrogen resistance protein 1
*NEK10*: NIMA related kinase 10
*CCND3*: Cyclin D3
*CCNA2*: Cyclin A2
*RBM38*: RNA binding motif protein 38
