# Supplementary material for: Genetic colocalization atlas points to common regulatory sites and genes for hematopoietic traits and hematopoietic contributions to disease phenotypes
Source: BMC Med Genomics. 2020 Jun 29;13:89. doi: 10.1186/s12920-020-00742-9 (PMC7325014; doi:10.1186/s12920-020-00742-9)
Supplement: Supplementary file 2 — Additional file 2: Figure S1. Colocalization between blood traits with limited genetic relatedness reflects hematopoietic lineage relationships. The 17 traits analyzed were pruned for genetic correlation (rg) < 0.8. a Number of traits identified at each colocalization site (max = 12). b Heat map depicting percent overlap at colocalization sites between each hematopoietic trait pair. In each box, the number of sites where the row-specified trait and column-specified trait colocalized was normalized to the total number of colocalization sites for the ‘row trait’. For this reason, the heat map is asymmetric. Color scale represents the proportion of loci where each pair of traits colocalized. To the left of the heat map, hierarchical clustering accurately segregated red cell, platelet, and white cell traits in general agreement with blood lineage relationships. c Degree of colocalization (% overlap) generally reflects genetic correlation between trait pairs. Shaded area depicts the 95% prediction interval, with gray line at mean. Colored spots highlight trait pairs outside the 95% prediction interval that included 2 platelet traits (purple) or 2 red blood cell traits (red). Exemplary trait pairs are labeled. Plt, platelet count. Mpv, mean platelet volume. Pdw, platelet distribution width. Rdw, red blood cell distribution width. Mchc, mean corpuscular hemoglobin content. Mcv, mean red cell corpuscular volume. Figure S2. Genetic colocalization among traits with limited genetic correlation reveals shared regulatory loci and implicates causal genes underlying genetic associations between hematopoietic traits and disease end-points. The 45 traits analyzed were pruned for genetic correlation (rg) < 0.8. a Number of traits identified at each colocalization site (max = 14). b Heat map depicting percent overlap at colocalization sites between each trait pair. In each box, the number of sites where the row-specified trait and column-specified trait colocalized was normalized to t [file 12920_2020_742_MOESM2_ESM.docx]

**Genetic colocalization atlas points to common regulatory sites and genes for hematopoietic traits and hematopoietic contributions to disease phenotypes**

Thom CS^1,2,3,4^, Voight BF^2,3,4^*

^1^Division of Neonatology, Children’s Hospital of Philadelphia, Philadelphia, PA, USA

^2^Department of Systems Pharmacology and Translational Therapeutics, Perelman School of Medicine, University of Pennsylvania, Philadelphia, PA, USA

^3^Department of Genetics, Perelman School of Medicine, University of Pennsylvania, Philadelphia, PA, USA

^4^Institute of Translational Medicine and Therapeutics, University of Pennsylvania, PA, USA

*Corresponding Author:

Benjamin F Voight

University of Pennsylvania Perelman School of Medicine

3400 Civic Center Blvd

10-126 Smilow Center for Translational Research

Philadelphia, PA 19104

bvoight@pennmedicine.upenn.edu

215-746-8083

**Supplemental Information**

**
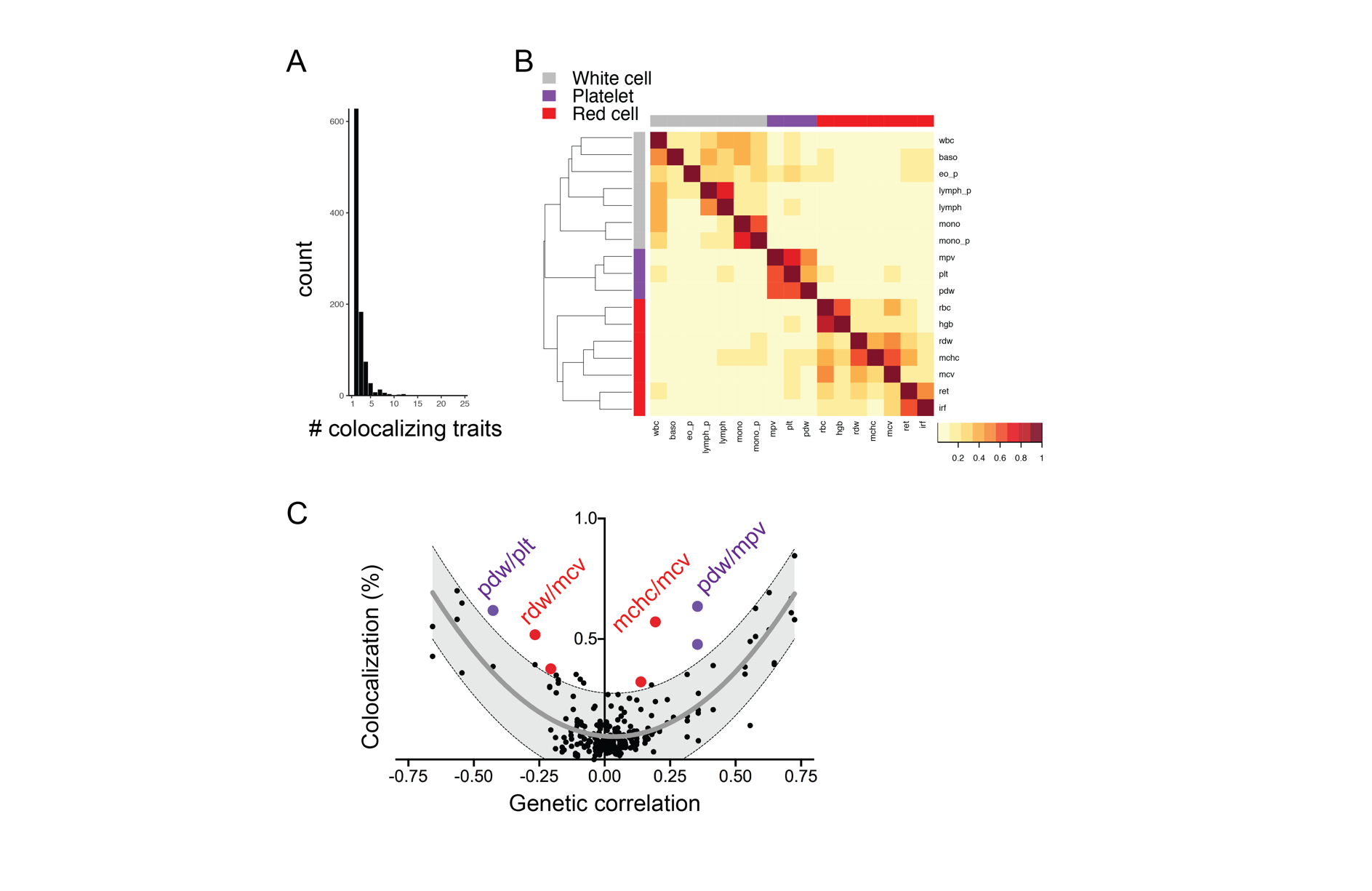
**

**Figure S1. Colocalization between blood traits with limited genetic relatedness reflects hematopoietic lineage relationships.**

The 17 traits analyzed were pruned for genetic correlation (r_g_) <0.8. **a** Number of traits identified at each colocalization site (max = 12). **b** Heat map depicting percent overlap at colocalization sites between each hematopoietic trait pair. In each box, the number of sites where the row-specified trait and column-specified trait colocalized was normalized to the total number of colocalization sites for the ‘row trait’. For this reason, the heat map is asymmetric. Color scale represents the proportion of loci where each pair of traits colocalized. To the left of the heat map, hierarchical clustering accurately segregated red cell, platelet, and white cell traits in general agreement with blood lineage relationships. **c** Degree of colocalization (% overlap) generally reflects genetic correlation between trait pairs. Shaded area depicts the 95% prediction interval, with gray line at mean. Colored spots highlight trait pairs outside the 95% prediction interval that included 2 platelet traits (purple •) or 2 red blood cell traits (red •). Exemplary trait pairs are labeled. plt, platelet count. mpv, mean platelet volume. pdw, platelet distribution width. rdw, red blood cell distribution width. mchc, mean corpuscular hemoglobin content. mcv, mean red cell corpuscular volume.


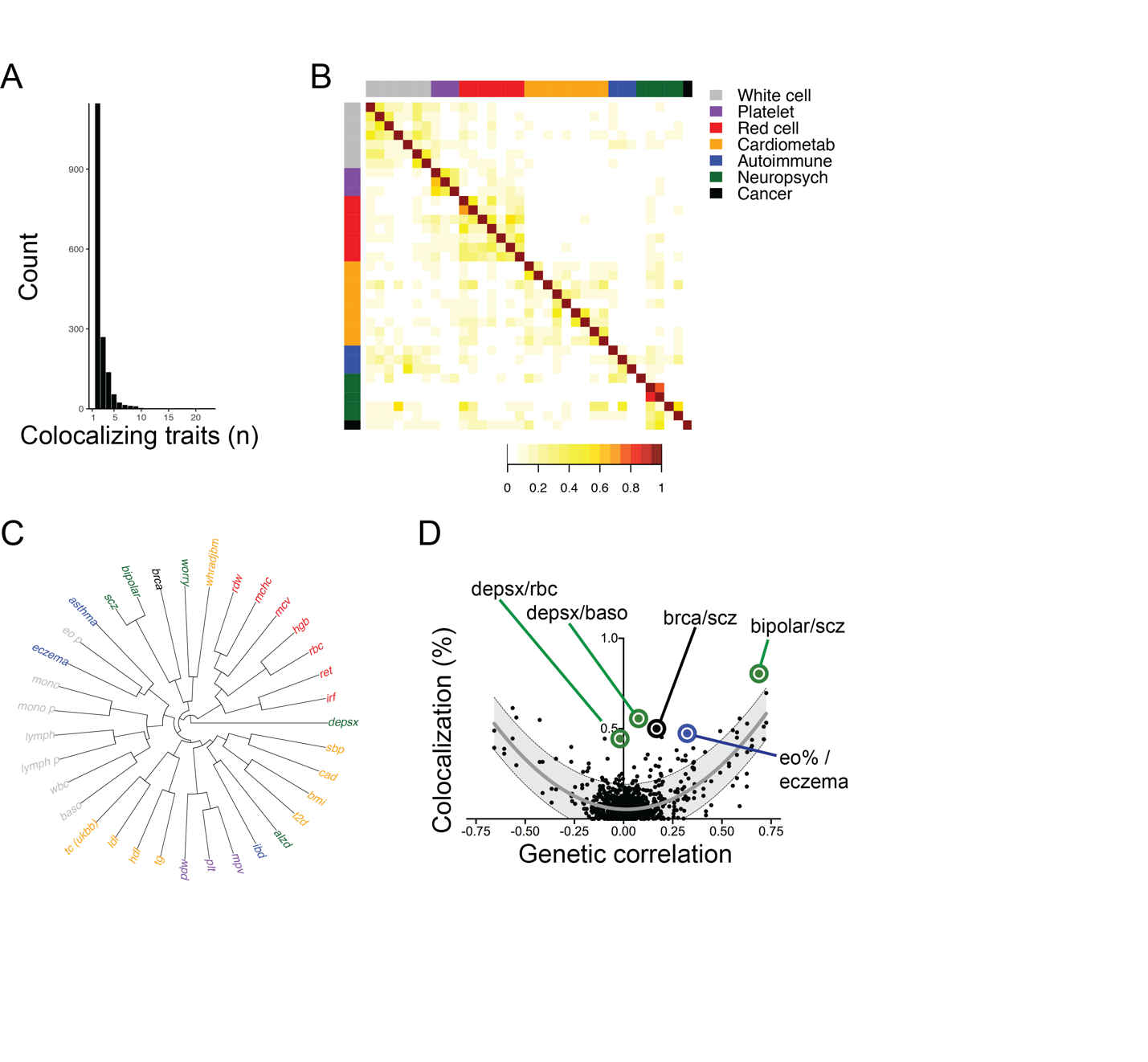


**Figure S2. Genetic colocalization among traits with limited genetic correlation reveals shared regulatory loci and implicates causal genes underlying genetic associations between hematopoietic traits and disease end-points.**

The 45 traits analyzed were pruned for genetic correlation (r_g_) <0.8. **a** Number of traits identified at each colocalization site (max = 14). **b** Heat map depicting percent overlap at colocalization sites between each trait pair. In each box, the number of sites where the row-specified trait and column-specified trait colocalized was normalized to the total number of colocalization sites for the ‘row trait’. For this reason, the heat map is asymmetric. **c** Hierarchical clustering based on colocalization results associates related traits, which are color coded according to the key in part **b**. **d** Degree of colocalization (% overlap) reflects genetic correlation between trait pairs. Shaded area depicts the 95% prediction interval, with gray line at mean. Exemplary trait pairs are circled. depsx, depressive symptoms. rbc, red blood cell count. baso, basophil cell count. brca, breast cancer. scz, schizophrenia. eo%, eosinophil percentage of white blood cells.
